# A case of suspected propofol infusion syndrome after abdominal aortic aneurysm surgery

**DOI:** 10.1186/s40792-020-00946-2

**Published:** 2020-07-31

**Authors:** Atsushi Guntani, Ryosuke Yoshiga, Shinsuke Mii

**Affiliations:** Department of Vascular Surgery, Saiseikai Yahata General Hospital, 5-9-27 Haruno-machi, Yahatahigashi-ku, Kitakyushu, 805-8527 Japan

**Keywords:** Propofol infusion syndrome, Abdominal aortic aneurysm, Rhabdomyolysis

## Abstract

**Background:**

Propofol infusion syndrome (PRIS) is a rare but potentially lethal side effect during propofol administration.

**Case presentation:**

The patient was scheduled for abdominal aortic aneurysm resection and reconstruction. Propofol used during sedation for ventilation after the surgery-induced rhabdomyolysis, heart failure, and renal failure. Discontinuation of propofol administration led to a dramatic improvement in the fatal symptoms, resulting in a diagnosis of PRIS.

**Conclusions:**

We herein report a rare case of a PRIS during sedation in the intensive care unit after abdominal aortic aneurysm surgery. Physicians using propofol should therefore be aware of the potential risk of PRIS.

## Background

Propofol infusion syndrome (PRIS) is a rare but potentially lethal side effect of propofol, which is commonly used in anesthesia and in intensive care for sedation. It is characterize by symptoms such as metabolic acidosis, rhabdomyolysis, arrhythmia, renal failure, and myocardial failure. Several cases of severe metabolic acidosis during propofol infusion have been reported previously, and Bray proposed the concept of PRIS for such a clinical state associated with propofol infusions in 1998 [1–4].

We herein report a rare case considered to be PRIS after abdominal aortic aneurysm surgery.

## Case presentation

A 70-year-old female patient was scheduled for abdominal aortic aneurysm resection and reconstruction. She had undergone general anesthesia for surgery of purulent cervical spondylitis about 6 years ago without any adverse event. The height of the patient was 142 cm, and the weight was 54 kg.

A catheter was inserted into the thoracic vertebrae 9–10 epidural interspace to enable analgesia prior to sedation using propofol. Anesthesia was induced with continuously injected remifentanil at a rate of 0.15–0.3 mg/kg/h, and propofol was started intravenously at a target plasma concentration of 3 μg/ml using a target-controlled infusion (TCI) pump, followed by 40 mg of rocuronium bromide for endotracheal intubation. Anesthesia was maintained with 70% nitrous oxide, 30% oxygen, and propofol continuously injected at a target plasma concentration of 1–2 μg/ml.

The abdominal aortic aneurysm, which measured 4.9 cm in diameter, was observed from the infrarenal artery to the level of bifurcation of the inferior mesenteric artery (IMA), while the renal artery and iliac artery were not clamped. The peripheral anastomosis to the aorta was possible above the IMA. The operation time was extended significantly beyond the scheduled time in order to control bleeding due to a lumbar vein injury, and the anesthesia lasted 5 h 38 min. Total blood loss was 3424 ml, 1159 ml in the blood loss was returned by the autologous blood collection device, and 6 units of red cell concentrates mannitol-adenine-phosphate were used. However, abdominal aortic aneurysm resection and reconstruction were performed routinely, and the patient’s hemodynamic condition was stable throughout the anesthesia course. We confirmed adequate arousal after the surgery, and the endotracheal tube was removed in the operating room, after which the patient was transferred to the recovery room.

On the second post-operative day, oliguria was prolonged, and her respiratory frequency exceeded 45 times with respiratory distress appearing due to pulmonary edema. Therefore, we decided that re-intubation and a ventilator were necessary, and propofol was used for sedation. Prior to endotracheal intubation, 30 mg of propofol was injected, and sedation was maintained with propofol at a rate of 50 mg/h using an infusion pump instead of a TCI pump.

From the following day, a fever of 39.7 °C and a decrease in blood pressure appeared. Blood tests showed that platelets had dropped to 26,000/μl, and creatinine phosphokinase (CPK) had risen to 57220 U/l. A blood gas analysis during propofol infusion showed pH 7.40, PCO_2_ 23.4 mmHg, HCO_3_ 14.2 mmol/L, base excess − 9.0 mmol/L, anion gap 22.0 mmol/L, and lactate 1.5 mmol/L. No significant elevation of lactate or acidosis was observed. Metabolic acidosis may have been compensated by mechanical ventilation (Table 1).

**Table 1:** Clinical course and laboratory data

Factor	OR	POD1	POD2	POD3	POD4	POD5	POD6	POD7	POD8	POD9	POD10	POD11	POD13
pH	7.52	7.32	7.43	7.54	7.53	7.55	7.45	7.4	7.44	7.46	7.45	7.44	7.48
PaCO2 (mmHg)	29	49.5	31.6	26.8	28.5	27.4	26.3	23.4	28	27.3	29.3	29.5	32.9
PaO2 (mmHg)	175	75.7	121.4	88.8	87.9	96.7	102.9	167.8	83.7	109.2	115.6	105.7	139.4
HCO3 (mmol/L)	23.7	24.9	20.6	22.2	23.1	23.2	17.7	14.2	18.5	19.1	20.1	19.5	23.7
Base excess (mmol/L)	1.5	− 1.6	− 2.7	1	1.2	1.7	− 4.9	− 9	− 4.6	− 3.8	− 3.2	− 3.8	0.4
Anion gap (mmol/L)		13.8	22.7	20.3	13.9	15.7	18.1	22	21.5	17.9	15.5	16.5	10.7
Lactate (mmol/L)		2	1.4	2.1	2.3	1.7	2	1.5	2.3	1.1	1.2	1.1	1.1
Creatinine phosphokinase (×1000 U/L)	0.108	0.428		0.53	0.286	5.18	46.25	57.22	48.39	26.47	7.294		1.159
Body temperature (°C)	35.5	37.8	38.7	39.7	40.2	41.1	39.7	39.6	36.8	36.4	36.2	36.4	36.2

*OR* operating room, *POD* post-operative day

We suspected intestinal ischemia and severe infection, and immediately started treatment for disseminated intravascular coagulation, stopping using epidural anesthesia. Colonoscopy revealed mild ulcers but no intestinal necrosis, myocardial infarction was denied from echocardiographic findings, and skeletal muscle necrosis was negative according to clinical findings. Thereafter, a fever of 41.1 °C and high CPK persisted, and her hemodynamics were disrupted, so we finally stopped propofol infusion to maintain her blood pressure. Continuous hemodiafiltration (CHDF) was started for the oliguria due to deterioration of the renal function.

**Fig. 1:**
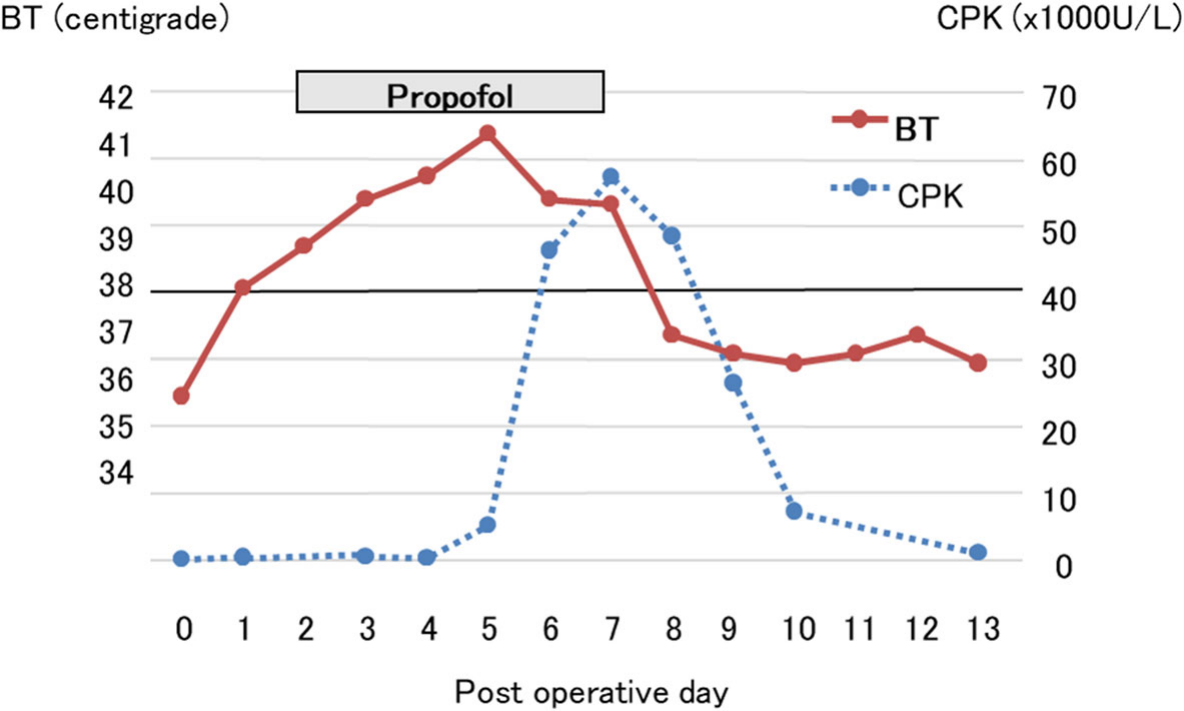
The figure showed that the clinical course of propofol infusion, body temperature (BT), and CPK decreased immediately after the discontinuation of propofol

From the day after the propofol infusion was stopped, the fever decreased to 36.8 °C, CPK started to decease, and the hemodynamics improved dramatically (Fig. 1). Two weeks later, her respiratory condition improved, and the ventilator was able to be removed. The urine volume and renal function had been stable, and hemodialysis was able to be discontinued. However, the patient suffered from prolonged consciousness disorder. Neither computed tomography (CT) nor magnetic resonance imaging (MRI) of the brain revealed any particular lesions causing prolong consciousness disorder other than a small area of cerebral infarction in the subacute phase. Concerning the continuation of consciousness disorder, an electroencephalogram was conducted, which suggested the possibility of seizures; however, the details were unclear. After two more weeks, the level of consciousness gradually improved, and speech and spontaneous movement became apparent.

After normalization of CPK and improvement of the general condition, re-examinations were performed, including CT and MRI; however, no disease other than PRIS causing an increase in CPK or a disruption of hemodynamics was suspected. Immediately after the injection of propofol was stopped, her condition improved, which eventually prompted our diagnosis.

Ten months after the operation, the patient’s communication level had improved, but hemiplegia remained due to cerebral infarction and muscle weakness due to disuse, so she was transferred to a rehabilitation hospital for functional recovery.

## Discussion

Although the symptoms of PRIS were initially reported to occur in children, according to the summary of recent case reports, they are more likely to be seen in adult or elderly patients and may occur even after a short duration of infusion and with usual doses of propofol [5]. Since there are no convincing symptoms and no clear diagnostic criteria of PRIS, the diagnosis may be challenging. Details of the pathogenesis of PRIS are unknown, but enhanced lipolysis, impaired fatty acid oxidation, and mitochondrial dysfunction have been suggested. PRIS presents with various organ disorders, including impairment of the cardiovascular, hepatic, and skeletal muscular systems, renal damage, and metabolic acidosis. In addition, the overall mortality of PRIS is reported to be 18% in these situations [6, 7].

In the literature, a multivariate analysis showed that, after adjusting for covariates, there are only a few independent predictors of death. The most important predictor seems to be the cumulative dose of propofol, being represented by both the mean infusion rate and the duration of infusion. On the other hand, cytotoxicity has been reported to occur at clinically relevant concentrations of propofol, and clinical studies have reported that PRIS may occur even at low doses and short durations of administration [8]. In the present case, the recommended dosage of propofol using a TCI pump was used during the surgery, and the total amount of propofol used during surgery was 1000 mg. A total of 2900 mg of propofol was used in the intensive care unit during the 5-day stay. The cumulative dose of propofol and the duration of infusion were rather high but still within the recommended usage range (0.3–3.0 mg/kg/h or less, usually for less than 7 days for sedation of adult patients [9, 10]).

PRIS in the present patient affected the cardiovascular system and induced cardiogenic shock. At the same time, the CPK value was abnormally high, suggesting skeletal muscle or myocardial damage or gastrointestinal necrosis. Echocardiography showed reduced myocardial motility, reflecting shock status, but this finding could not explain the extraordinary increase in CPK. Colonoscopy confirmed no gastrointestinal necrosis. Skeletal muscle damage might have been caused by rhabdomyolysis, in fact, by which the patient was complicated with acute kidney injury requiring CHDF for several days.

Diseases associated with a high fever and rhabdomyolysis that should be differentiated are malignant hyperthermia and malignant syndrome. Malignant hyperthermia causes hypercontraction of the skeletal muscle due to an inherited disorder of calcium metabolism triggered by volatile anesthetic agents, especially halothane, and depolarizing muscle relaxants, such as succinylcholine. Our case was not administered any volatile anesthetic agents and had no such familial history, and no hypercapnia and muscular rigidity, which commonly occur during malignant hyperthermia, were observed. Furthermore, the present event did not resemble the typical course of malignant hyperthermia, since it occurred in the intensive care unit after surgery; however, the occurrence of malignant hyperthermia due to propofol has also been reported and cannot be completely denied. Therefore, the administration of dantrolene sodium might have been considered as a diagnostic treatment. The possibility of malignant syndrome, by contrast, was negative, as she had no history of antipsychotics or psychotropic medication [11–13]. The dramatic improvement in the general condition immediately after the discontinuation of propofol was an important point leading to the differential diagnosis of PRIS.

## Conclusion

Physicians using propofol should be aware of the potential risk of PRIS. If a patient receiving propofol develops unexplained symptoms, such as lactic acidosis, rhabdomyolysis, or renal failure, propofol infusion should be stopped immediately, and other critical illnesses should be distinguished.

## Data Availability

All datasets supporting the conclusions of this article are included in this published article.

## Abbreviations

PRIS: Propofol infusion syndrome
TCI: Target controlled infusion
IMA: Inferior mesenteric artery
CPK: Creatinine phosphokinase
CHDF: Continuous hemodiafiltration
CT: Computed tomography
MRI: Magnetic resonance imaging

## References

[1] Kam PCA, Cardone D (2007). Propofol infusion syndrome. Anaesthesia.

[2] Parke TJ, Stevens JE, Rice ASC, Greenaway CL, Bray RJ, Smith PJ (1992). Metabolic acidosis and fatal myocardial failure after propofol infusion in children: five case reports. British Medical Journal.

[3] Burow BK, Johnson ME, Packer DL (2004). Metabolic acidosis associated with propofol in the absence of other causative factors. Anesthesiology.

[4] Bray RJ (1998). Propofol infusion syndrome in children. Paediatr Anaesth.

[5] Krajčová A, Waldauf P, Anděl M, Duška F (2015). Propofol infusion syndrome: a structured review of experimental studies and 153 published case reports. Critical Care.

[6] Sumi C, Okamoto A, Tanaka H, Nishi K, Kusunoki M, Shoji T, et al. Propofol induces a metabolic switch to glycolysis and cell death in a mitochondrial electron transport chain-dependent manner. PLOS ONE. 2018; 10.1371/journal.pone.0192796. 10.1371/journal.pone.0192796 PMC5813975 29447230

[7] Mirrakhimov AE, Voore P, Halytskyy O, Khan M, Ali AM. Propofol Infusion Syndrome in Adults: A Clinical Update. Critical Care Research and Practice. 2015; 10.1155/2015/260385. 10.1155/2015/260385 PMC4410753 25954513

[8] Roberts RJ, Barletta JF, Fong JJ, Schumaker G, Kuper PJ, Papadopoulos S (2009). Incidence of propofol-related infusion syndrome in critically ill adults: a prospective, multicenter study. Critical Care.

[9] Liolios A, Gue’rit JM, Scholtes JL, Raftopoulos C, Hantson P (2005). Propofol Infusion Syndrome Associated with Short-Term Large-Dose Infusion During Surgical Anesthesia in an Adult. Anesth Analg.

[10] Ichikawa T, Okuyama K, Kamata K, Masui K, Ozaki M. Suspected propofol infusion syndrome during normal targeted propofol concentration. J Anesth. 2020; 10.1007/s00540-020-02773-z. 10.1007/s00540-020-02773-z 32222909

[11] Gulabani M, Gurha P, Ahmad S, Dass P (2014). Intra-operative post-induction hyperthermia, possibly malignant hyperthermia: Anesthetic implications, challenges and management. J Anaesthesiol Clin Pharmacol.

[12] Rosenberg MB (1991). Propofol for anesthesia in a patient susceptible to malignant hyperthermia. Anesth Prog.

[13] Inada H, Jinno S, Kohase H, Fukayama H, Umino M (2005). Postoperative hyperthermia of unknown origin treated with dantrolene sodium. Anesth Prog.

